# An Unusual Lingual Ulcerated Capillary Angioma After Dressmaker Needle Trauma, Above Migratory Glossitis Lesion, in a Patient With a History of Cancer: A Case Report

**DOI:** 10.1155/crid/9921608

**Published:** 2025-05-06

**Authors:** Cinzia Casu, Andrea Butera, Clara Gerosa, Andrea Scribante, Martina Salvatorina Murgia, Germano Orrù

**Affiliations:** ^1^Oral Biotechnology Laboratory (OBL), Department of Molecular Biology, University of Cagliari, Cagliari, Italy; ^2^Section of Dentistry, Department of Clinical, Surgical, Diagnostic and Pediatric Sciences, University of Pavia, Pavia, Italy; ^3^Unit of Pathology, Department of Medical Sciences and Public Health, University of Cagliari, Cagliari, Italy; ^4^Department of Surgical Science, University of Cagliari, Cagliari, Italy

**Keywords:** capillary hemangioma, miRNA, molecular biology evaluation, oral angioma, oral benign tumor, oral metastasis, oral vascular lesions

## Abstract

Vascular malformations are relatively common benign proliferative lesions of vascular and connective tissue origin that can present in oral regions, such as on the tongue. Etiological factors include genetic mutations or molecular changes related to syndromes, trauma, vascular wall resistance to blood flow, and may form part of other systemic diseases. Clinically, they can be extremely heterogeneous, and they can also cause important diagnostic doubts. Migratory glossitis is a very common oral condition in the general population, linked to immunological factors, sometimes connected with intestinal health problems. The aim of this work is to report an atypical case of ulcerated angioma on the tongue and the contemporary presence of migratory glossitis. A 54-year-old Caucasian female with a history of an intestinal cancer presented a particular exophytic lesion on the tip of the tongue where the hypothetical diagnosis of oral metastasis had been included, also for the rapid onset of the oral lesion. The dressmaker's needle trauma related to the patient's work activity could have been the triggering factor for the appearance of the neoformation. The diagnosis after histological evaluation was ulcerated capillary angioma. Due to the unusual presentation of this type of lesion, it is needed to reconsider current knowledge on the etiopathogenesis of vascular lesions and its clinical inclusion in the differential diagnosis.

## 1. Introduction

Vascular malformations are benign lesions characterized by the proliferation of blood vessels, relatively common in the head and neck region [1, 2]. Their classification is much debated; in fact, the International Society for the Study of Vascular Anomalies (ISSVA) classified vascular anomalies into two large groups: vascular tumors (hemangiomas) and vascular malformations, based on the clinical and histopathological characteristics of the lesions. The latest update in their ISSVA classification, in 2023, considers also etiological factors [3]. Vascular tumors are divided into benign, locally aggressive/borderline, and malignant. The vascular malformations can be simple, combined, those of named vessels, and those associated with other anomalies. Etiological factors include genetic mutations or molecular changes related to syndromes, trauma, vascular wall resistance to blood flow, and may form part of other systemic diseases [2, 3]. Most of these lesions are congenital or occur at a young age and may show limited growth potential. Some lesions regress spontaneously; others require treatment [2]. This type of lesion consists of an exuberant proliferation of connective tissue and blood vessels that occurs in response to a known stimulus or chronic irritating factors, including bacterial plaque and calculus [4]. The most affected oral regions are the lips, tongue, buccal mucosa, gums, and palate [2–8]. Clinically, they appear as raised, lumpy, dark-colored lesions, sometimes ulcerated, soft, or soft-elastic on palpation [6].

In a Brazilian study, the authors who conducted a survey of patients seen in an oral pathology department found that oral hemangioma represented 14.3% of oral vascular lesions (mainly seen in children), while vascular malformations represented 20% of these, while varicose veins were even more frequent [9].

Oral hemangiomas represent 60% of vascular lesions in the head and neck district but are rare on the tongue [10].

Geographic tongue is a recurring chronic inflammatory condition of the oral cavity of unknown aetiology, characterized by very superficial erosions on the dorsum and lingual margins, sometimes circumscribed by white circles. These erosions, mostly asymptomatic, tend to move over the course of days or weeks; in fact, this condition, which afflicts up to 2%–3% of the population, is also called benign migratory glossitis. It can often be associated with systemic manifestations such as psoriasis or gastrointestinal disorders and can sometimes present itself as the first manifestation of the systemic picture [11, 12].

Consequently, the aim of this report is to describe a particular acquired vascular lesion on the tongue affected also by geographic tongue, in an adult patient with a history of cancer with a focus on the diagnostic process. Correlations between benign migratory glossitis, oral vascular lesions of the tongue, and development of intestinal carcinoma have not been found in scientific literature. We have not found other cases documented in the literature of the copresence of a lingual vascular lesion and benign migratory glossitis, and the particular clinical manifestation of the vascular lesion presented extremely varied aspects that made the diagnostic process very difficult, also taking into consideration any oral metastasis.

## 2. Case Report

A 54-year-old white female came to our department for the appearance of a lesion on the tip of the tongue. The patient did not report pain and developed the lesion in approximately 4 weeks. Her medical history included an intestinal adenocarcinoma treated with radio (20 sessions in few weeks) and chemotherapy (5-fluorouracil) 5 years before. No other health problems, allergies, or current medication intake were reported, but a family predisposition (mother in particular) to the development of benign acquired vascular malformations was reported. She reported having a small trauma with a dressmaker's needle on the tongue 1 month before and also that it is not the first time that during her work activity she had this type of needle trauma.

**Figure 1:**
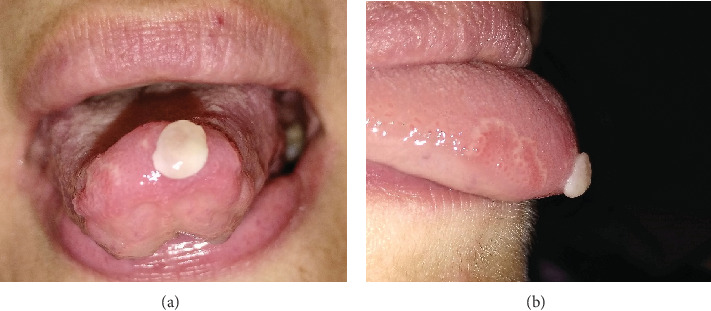
(a) Frontal view of the neoformation on the tip of the tongue. (b) Lateral view in which the benign migratory glossitis is clearly visible.

Clinical oral examination showed a solitary, well-circumscribed white exophytic lesion, measuring approximately 1 cm in diameter with soft-elastic texture on palpation (Figure 1a,b). In addition, erosive lesions on the anterior third of the dorsum on the right, on the posterior third of the dorsum on the left, and on the left lingual margin, surrounded by a white ring, were detected. These conditions were attributable to geographic tongue and included the area where the neoformation arose, but they did not appear related to the onset of the exophytic lesion. The lesion appeared to be based on a sessile implant on the underlying lingual mucosa but with a diameter smaller than the maximum diameter of the lesion itself. The edges were regular and not thickened to palpation, while the surrounding dorsum of the tongue tissue appeared overall normochromatic.

Palpable neck lymphadenopathy was not detected. Radiological investigations were not performed because the mass was palpable and located in the soft tissue. Due to her medical history, metastases were included in the differential diagnosis, for which an incisional biopsy for histological examination was planned after 2 weeks, only because the event occurred during holidays (in August) and it is not possible to perform the biopsy earlier. Following the appearance mode of the lesion and the reactive etiology hypothesis, fibroma, peripheral giant cell granuloma, and pyogenic granuloma were included in the differential diagnosis.

The clinical presentation—an asymptomatic, nonremovable white pseudomembrane covering the lesion—could suggest a keratinization process as a response to chronic trauma such as that reported by the patient. No other signs of infection were detected; the exophytic presentation (not a raised plaque) excluded an oral leucoplakia or a lichenoid lesion. Despite the strong family predisposition, especially several skin ruby hemangiomas, the color of the lesion did not suggest a vascular lesion; however, it was included among the possible causes.

During this period, the patient traumatized this area by biting the tongue and so, an excisional biopsy was immediately performed by the oral maxillofacial surgery department. In fact, since a good part of the lesion had detached from the rest of the underlying mucosal tissue following a bite trauma, it was decided to remove it all to ensure a better treatment and prognosis for the patient.

The operation was performed promptly, a few hours after the trauma which caused significant bleeding, despite the patient not having any problems with impaired coagulation. The operation was performed with a cold blade, after local anesthesia with a 1.8-mL tubular vial of articaine with adrenaline 1:100,000. The suture was performed with a technique of interrupted stitches and with additional continuous suture with 3.0 silk threads to prevent bleeding. Silk was used because it was considered more resistant than other absorbable materials both to contain the residual flaps after removal of the lesion and to hermetically seal the tissue and prevent blood leakage even postsurgery. The sample was placed in a sterile container with 10% buffered paraformaldehyde and sent to the pathological anatomy laboratory. The suture was removed, again in hospital, 15 days later.

**Figure 2:**
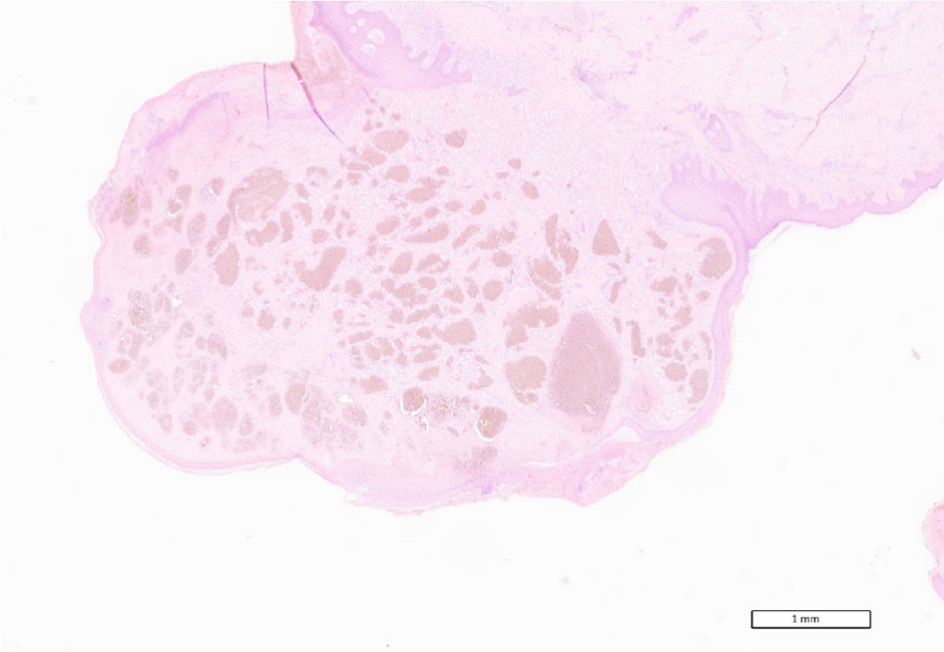
Histological aspects of the neoformation in the tip of the tongue, EE4X.

The histopathological analysis indicated a diagnosis of an unusual case of ulcerated capillary angioma. The histological slide shows capillary angiomatous hyperproliferation with ulcerative aspects (Figure 2). Follow-up examinations at 1 and 4 weeks and 6 months showed mucosal integrity and no sign of recurrence. Following the histopathological diagnosis, and questioning the patient, it emerged that she used to sew for many hours a day. Therefore, it is supposed that the microtrauma which caused the vascular injury was the constant cutting and moistening of the wire through the anterior teeth and the tip of the tongue.

## 3. Discussion

Numerous aspects emerge from this case that deserve to be discussed. The differential diagnosis of this tongue exophytic lesion includes oral metastasis, oral carcinoma, and oral inflammatory-traumatic lesion.

### 3.1. Correlation With Oral Metastasis

First of all, lesions that may seem benign must always be investigated in the context of the patient's medical history.

In fact, although oral metastases are rare, they should never be excluded in the differential diagnosis, especially in patients with previous cancer [13, 14]. Kaplan et al. found that metastases in the oral cavity are distributed almost equally between the soft tissues and the maxillary bones (48.3% and 51.7%, respectively) [15]; other authors reported that jaw bones are more involved than oral soft tissues [16, 17]. The most common oral soft tissue metastases sites are the gum followed by the tongue accounting for 54% and 23% of all cases, respectively [16]. The most frequent malignancies which can metastasize in the oral cavity are breast (25.8%), colorectal cancer (16.3%), lung (12%), and prostate (10%) [15]. Ho et al. found that in males [16], the second most common primary site was the colon (4 of 22, 18.2%); an oral metastasis from intestinal carcinoma represented 11% of cases of oral metastasis from all primary tumors, in a very recent work [17]. Clinically, lingual metastatic lesions appear as submucosal nodular masses [18] or as nodular exophytic lesions [19]. Tongue metastases usually are asymptomatic [18]. Pakfetrat et al. in their clinical experiences and review of the literature on the subject wrote that “an important characteristic of oral soft tissue metastatic lesions is that they can mimic the appearance of benign neoplastic, inflammatory, or reactive lesions, including pyogenic granuloma (PG), peripheral giant cell granuloma (PGCG), peripheral ossifying fibroma, and hemangiomas or vascular malformations” [17]. Hisberg et al. in their review found that the tongue, as a vascular tissue, can facilitate the uptake of tumor cells and for this reason is one of the most common metastases sites in oral soft tissues [20]. As regards the clinical features, our case reflects the main features of vascular lesion including the preference of female gender over male and the higher incidence among the Caucasian population than the others [21].

### 3.2. Development of Trauma-Related Malignant Lesions

Benign reactive lesions, where the pathogenic noxa is represented by chronic repeated trauma, can evolve into malignant conditions, even in patients without a history of oral cancer [13, 22]. In fact, a recent review on the subject revealed that chronic mechanical irritation could fasten the carcinogenesis process through the epigenetic changes inhibiting DNA reparation and could be a promoter, even if it is not the direct responsible for the genetic mutations. The areas in the oral cavity that are mainly traumatized are the tongue, such as in this case presented [22]. However, it is also true that it is much more common to find benign oral conditions in patients with a history of cancer rather than oral metastases from primary distant tumors [23].

### 3.3. Noninvasive Tools to Intercept Oral Malignancies

Recently, several researchers have discovered the important and noninvasive contribution of molecular biology in the interception of oral malignant lesions and distant metastases through the evaluation of the expression of salivary microRNAs (miRNAs). These are small noncoding RNA molecules, on average 15–20 nucleotides long, which are involved in the regulation of gene expression and which play an important role in various biological processes, released from tissues affected by a particular inflammatory or neoplastic pathology [24, 25]. The evaluation of these molecules both at the plasma level and even more so at the salivary level is arousing much interest from international scientific research, especially for their predictive and diagnostic value [26, 27]. In this case presented by us, the evaluation of the miRNA expression pattern would have been interesting to exclude an oral malignant lesion or a metastatic lesion from intestinal carcinoma through “liquid biopsy” using saliva.

### 3.4. Clinical Aspects of Capillary Angioma and Correlation With Dressmaker's Needle Trauma

Only one case of oral capillary angioma with clinical and histopathological features similar to this case has been found in the literature [4]. In this case, the lesion arose in a 10-year-old child in the adherent gingiva in the anterior maxillary region [4]. Capillary angiomas generally do not affect adjacent bones, and they can also be superficial, as in this case, or deep. Cases of intramuscular hemangiomas are described in the literature [28]. Cases of oral ulcerated capillary angioma, not in childhood patients, documented in literature are very few. Some authors reported 13 cases, mostly related to traumatic factors [29]. We have not found any work in scientific literature that reports the onset of an oral vascular lesion following trauma with a dressmaker's needle.

Capillary angiomas may be small, as in this case, but, increasing in volume, they can cause functional problems including chewing, phonetic and psychological difficulties, as well as causing bleeding if accidentally traumatized; for this reason, they often need to be removed [30].

### 3.5. Management of Similar Lesions

This type of acquired vascular neoformations should be treated with great caution due to the risk of profuse bleeding and treated or removed with instruments that can prevent this phenomenon: for example, through the use of diode lasers not only for the safety of surgical removal but also for photocoagulation and to perform low-level laser therapy (LLLT). Parameters successfully used are 980 nm, 1 W in gated pulse mode [31]; 980 nm in noncontact mode with a 300-nm fiber in continuous wave, power of 1.5 W [32], for photocoagulation; and 940 nm, 3.5 watts, a pulse length of 0.05 ms, and an interval of 0.20 ms by 400 nm [33] for laser ablation and 780 nm, 60 mW, and 3.0 J/cm^2^ for LLLT [31].

The correlation between migratory glossitis and endothelial proliferation is poor in the literature [34], and no data on the influence of the healing process after a surgical removal of an exophytic lesion is reported. LLLT has been shown to be effective in the management of symptomatic cases of benign migratory glossitis in a recent randomized clinical trial, performing sessions with a 660-nm diode laser with 2-min applications for each erosion [35].

### 3.6. Clinical Significance

This clinical case highlights three essential elements:

1. The anamnesis is a very important process, which often guides the clinician toward the diagnosis, especially when the clinical aspect is confusing.

2. The histological examination remains even today fundamental to establish with certainty the diagnosis, reducing the risk of underestimating or overestimating the lesions.

3. Mechanical trauma can sometimes lead to hyperproliferation of the endothelium and therefore generate extremely vascularized lesions.

No other reactive oral lesions due to this type of trauma have ever been documented. This may be the first case of ulcerated capillary angioma, in an adult, related to chronic dressmaker's needle trauma. Knowledge of the most oral soft tissue lesions is an essential component of daily clinical practice. Oral pathologies are relatively common entities, but some, as in this case, can cause significant diagnostic doubts. The collection of the medical history is fundamental, especially of the remote pathological one and of the patient's daily habits. However, as in this case, the histopathological examination is decisive. In the near future, the use of molecular biology through the analysis of specific salivary miRNAs could be important to exclude malignant lesions of the oral cavity even before histological evaluation.

## Data Availability

The data that support the findings are available within the manuscript.
